# Highly Sensitive NH_3_ Wireless Sensor Based on Ag-RGO Composite Operated at Room-temperature

**DOI:** 10.1038/s41598-019-46213-9

**Published:** 2019-07-09

**Authors:** Lei Zhang, Qiulin Tan, Hairong Kou, Dezhi Wu, Wendong Zhang, Jijun Xiong

**Affiliations:** 1 Key Laboratory of Instrumentation Science & Dynamic Measurement, Ministry of Education, North University of China, Tai Yuan, 030051 China; 2 Science and Technology on Electronic Test and Measurement Laboratory, North University of China, Tai Yuan, 030051 China; 3 Department of Mechanical & Electrical Engineering, Xiamen University, Xiamen, 361005 China

**Keywords:** Electronic properties and devices, Sensors and biosensors

## Abstract

The detection of ammonia (NH_3_) in low concentrations is very important in the chemical industry and for human health. In this paper, we present reduced graphene oxide (RGO) decorated with silver nanoparticles (AgNPs) as a sensing material for NH_3_. A simple, environmentally friendly, and cost-efficient green approach for the preparation of the sensing material is proposed. X-ray diffraction (XRD), Raman spectroscopy, and field emission scanning electron microscopy (FE-SEM) were used to analyze the crystalline structure, material composition, and surface appearance characteristics of the sensing material. By combining the material with a commercial near-field communication (NFC) tag, a wireless gas sensor was built. The enhanced NH_3_-sensing performance is mainly due to the synergistic effect between Ag and RGO. More specifically, AgNPs enhanced the adsorption capacity of RGO for NH_3_ electrons. The excellent performance of the sensor shows that it has potential for applications in food safety, environment, and human health monitoring.

## Introduction

As a poisonous gas, NH_3_ is produced in many industrial processes and the natural decomposition of organic matter. Long-term exposure to 35 ppm of NH_3_ is hazardous to human health^1^. Gas sensors play an important role in the detection of hazardous gases, inflammable gases, air pollution, human health and agricultural automation^2–6^. Various gas sensing technologies have been developed^7^, traditional gas sensors with metal compounds as sensing materials detect gases at high temperatures and show excellent performances^8^. However, there is more of a need to test gas sensing at room temperature^9^.

In recent years, nanostructures have received much attention owing to excellent performances in gas sensing. They possess nano-sized particles, uniform sizes, narrow distribution and ultra-high specific surface areas^10–13^. Many nanostructures, such as SnO_2_, ZnO, CuO, and Fe_2_O_3_ metal oxide semiconductors, have been used to detect poisonous, inflammable, and explosive gases^14–17^. However, their higher operating temperatures have limited the application of metal oxide sensors. Carbon materials such as carbon nanotube and graphene are proved to be ideal gas sensitive materials, which can work at room temperature. Due to the high specific surface area, carbon nanotube has been used for gas sensing widely^18–20^. Graphene possesses excellent electronic, thermal and robust mechanical properties, and has recently become an emerging two-dimensional (2D) material^21^. Many researchers have attempted to combine metal nanomaterials with carbon nanomaterials such as graphene and carbon nanotubes to make super-sensitive sensing materials^22–25^. It has potential in many applications, such as energy storage and sensing^26^, and graphene can be considered as the ideal electrode. Graphene is a typical 2D material, it contains a single-layer sheet of sp^2^-hybridized carbon atoms with zero band gap energy and possesses excellent electrical conductance^27^. It is usually characterized as a p-type semiconductor, mainly owing to the oxygen-containing functional groups and defects^24^. The edge of a graphene-based composite material forms a porous structure, which increases the specific surface area and leads to better sensor response. Owing to its large surface area, the material shows excellent electrical performance and possesses high electrical conductivity and ultra-fast electron mobility, and the specific surface area is approximately twice as large as that of carbon nanotubes^27^. In addition, combining gas sensing materials with wireless sensors improve the application of the sensors in wearable, health monitoring and environmental monitoring effectively. As the development of the artificial intelligence (AI) and internet of things (IOT), wireless sensors possess particular advantage than traditional wired sensors. Based on the near-field electromagnetic coupling, the sensor realizes remote sensing without power supply.

In this paper, we reported a flexible wireless sensor that can monitor gases at low concentrations (5 ppm) at room temperature (25 °C). The sensor is based on a near-field coupled electronic tag and uses RGO decorated with AgNPs as the gas-sensitive material. The reflectance of the sensor would change when NH_3_ gas molecules are absorbed by the AgNP-decorated RGO, since the impedance of the wireless sensor circuit would change. The results showed that the sensor has a fast response time and recovery time, and also good repeatability. In addition, the electrical characteristics of the sensor hardly changed after bending, which means that the sensor has potential application value in future wearable devices.

## Results

### Preparation of sensitive materials

**Figure 1:**
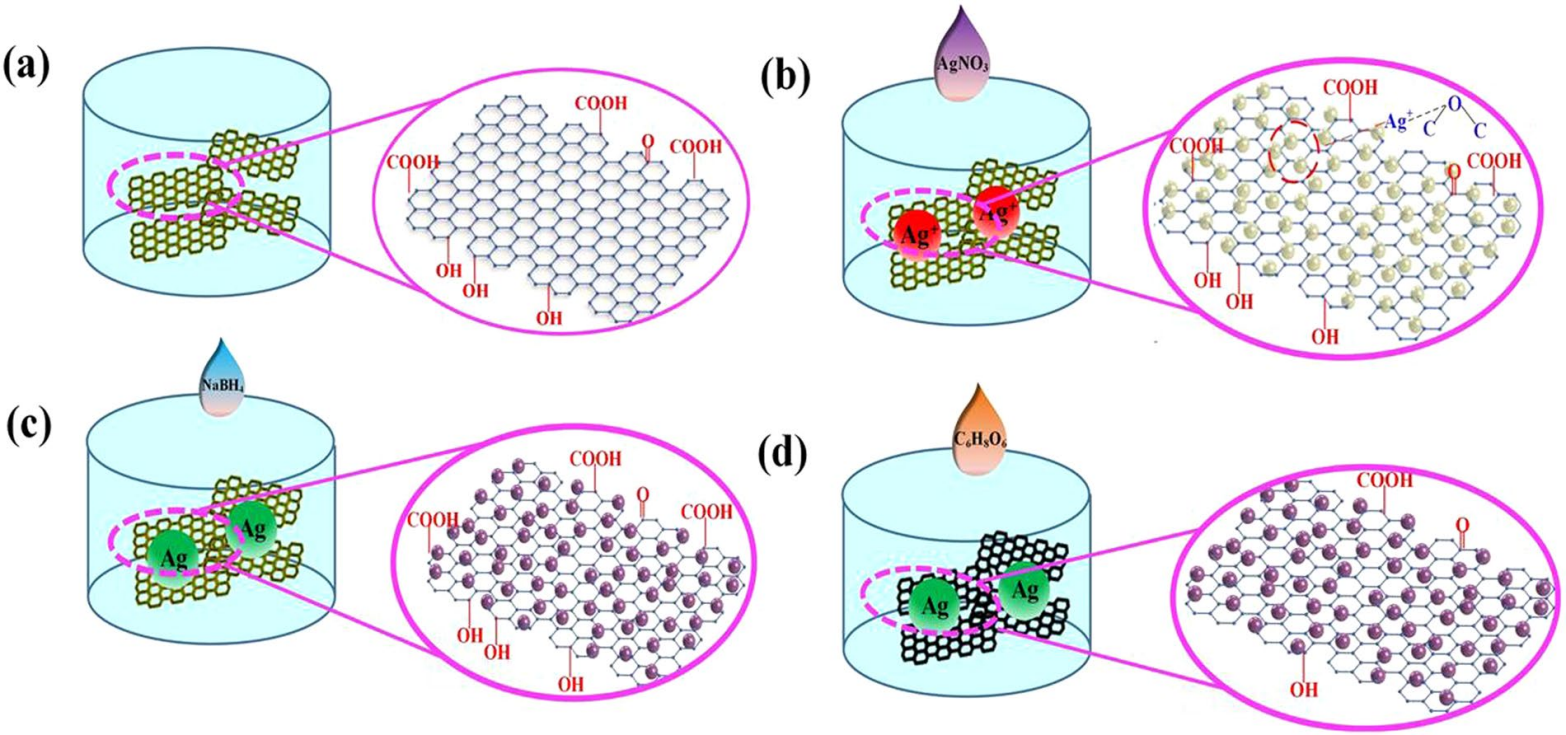
Preparation process of Ag-RGO. (**a**) Graphene oxide is dispersed in DI water and the schematic image of a graphene oxide (GO) sheet. (**b**) AgNO_3_ solution was added into the graphene oxide solution and the schematic image of Ag^+^-GO. (**c**) Silver ions were reduced to AgNPs by adding NaBH_4_ solution. (**d**) Ag-RGO was obtained by adding C_6_H_8_O_6_ solution.

To prepare NH_3_ sensing material, RGO was decorated using AgNPs. The preparation process involved the following steps, as shown in Fig. 1(a–d). First, the initial graphene oxide (GO) material was prepared from ultra-high-purity graphite powder (99%, Aldrich) according to a modified Hummers and Offman method^28^. Then, the prepared GO was dispersed in deionized water and the resulting mixture was ultrasonicated at 60 Hz for 1 h, to prepare a GO aqueous solution. Then, different volumes (0.1, 1, or 10 mL) of a 0.1 mol/L AgNO_3_ solution (Shuwan Chemical Industry, Dongguan) were added to the GO solution. There are many different functional groups on the surfaces of GO sheets, such as hydroxyl (-OH), carboxyl (-COOH), and epoxy groups (-CH(O)CH-)^29^.

When the AgNO_3_ solution was introduced into the GO solution, the positively charged Ag ions formed bonds with the partially negative oxygen atoms in the functional groups. When the Ag cations were adsorbed to the GO surface, NaBH_4_ (Damao Chemical Reagent Factory, Tianjin) dissolved in deionized water was introduced into the mixed solution as a reducing agent, and reduced the Ag cations from Ag^+^ to Ag. Consequently, an Ag-decorated GO sheet (Ag-GO) was produced. Then, ascorbic acid (Haibiao Science and Technology, Xiamen) was added to the Ag-GO solution as a reducing agent under vigorous stirring to convert the GO sheet to RGO. Thus, Ag-RGO as a gas-sensing material was prepared.

### Characterization of sensitive materials

**Figure 2:**
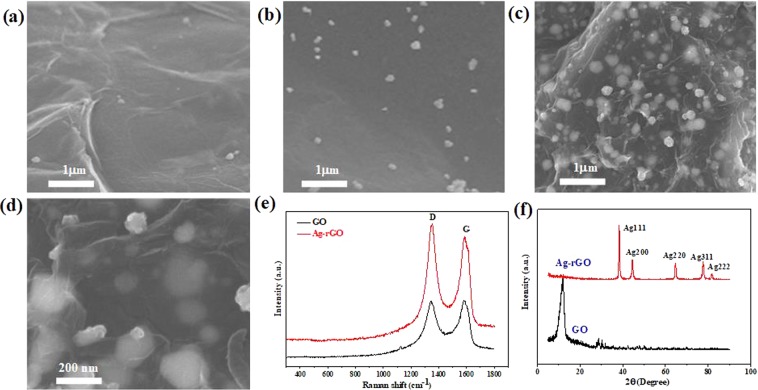
(**a**) SEM image of 0.1-Ag-RGO. (**b**) SEM image of 1-Ag-RGO. (**c**) SEM image of 10-Ag-RGO. (**d**) high-magnification image of 10-Ag-RGO. (**e**) Raman spectra of GO nanosheets before and after decoration with AgNPs. (**f**) X-ray diffraction (XRD) patterns of GO nanosheets before and after decoration with AgNPs.

The field emission scanning electron microscopy (FE-SEM) results for Ag-RGO are shown in Fig. 2. Figure 2(a–c) show the surface topographies of 0.1-Ag-RGO, 1-Ag-RGO, and 10-Ag-RGO, respectively, which reveal that there were different amounts of AgNPs on the surfaces of the RGO sheets. Figure 2(d) shows the appearance of the surface under high magnification. It can be concluded that as the number of Ag ions increases, the number of AgNPs on the graphene surface also increases, effectively increasing the specific surface area of graphene. Raman spectroscopy was used to analyze the vibrations and rotational information of the nanostructure. As shown in Fig. 2(e), there are two characteristic peaks of GO and Ag-RGO sheets. The characteristic peaks in the Raman spectrum of graphene are mainly D and G peaks. Figure 2(e) also shows that the graphene has defects owing to the oxidation, the D peak is at 1346 cm^−1^ and the G peak, which corresponds to optical E_2g_ phonons at the Brillouin zone center^30^, is at 1587 cm^−1^. The intensity ratio of the D and G peaks (I_D_/I_G_) of Ag-RGO changed noticeably from 0.994 to 1.103. Owing to the addition of the reducing agent, most of the functional groups on the GO surface were reduced, resulting in changes in I_D_/I_G_. X-ray diffraction (XRD, HAOYUAN DX-2007B) was used to analyze the nanocomposite components Fig. 2(f). The characteristic diffraction peaks at diffraction angles of 38.31°, 44.48°, 64.64°, and 81.27° correspond to the (111), (200), (220), and (311) crystal surfaces, respectively. The XRD results were consistent with the standard card of Ag (JCPDS 04-0783).

To be a good candidate as a gas sensing material, specific surface area is an important parameter. To determine specific surface area of Ag-RGO, the Brunauer–Emmett–Teller (BET) gas adsorption system was used. The specific surface area of Ag-RGO composite was measured by adsorption and desorption of nitrogen. The BET specific surface areas of the RGO, 0.1-Ag-RGO, 1-Ag-RGO and 10-Ag-RGO are calculated by BET method to be 436.6 m^2^g^−1^, 512 m^2^g^−1^, 568 m^2^g^−1^, 621.3 m^2^g^−1^ respectively. The specific surface area of sensing material increased with Ag nanoparticles loading.

### Gas sensing evaluation of Ag-RGO

**Figure 3:**
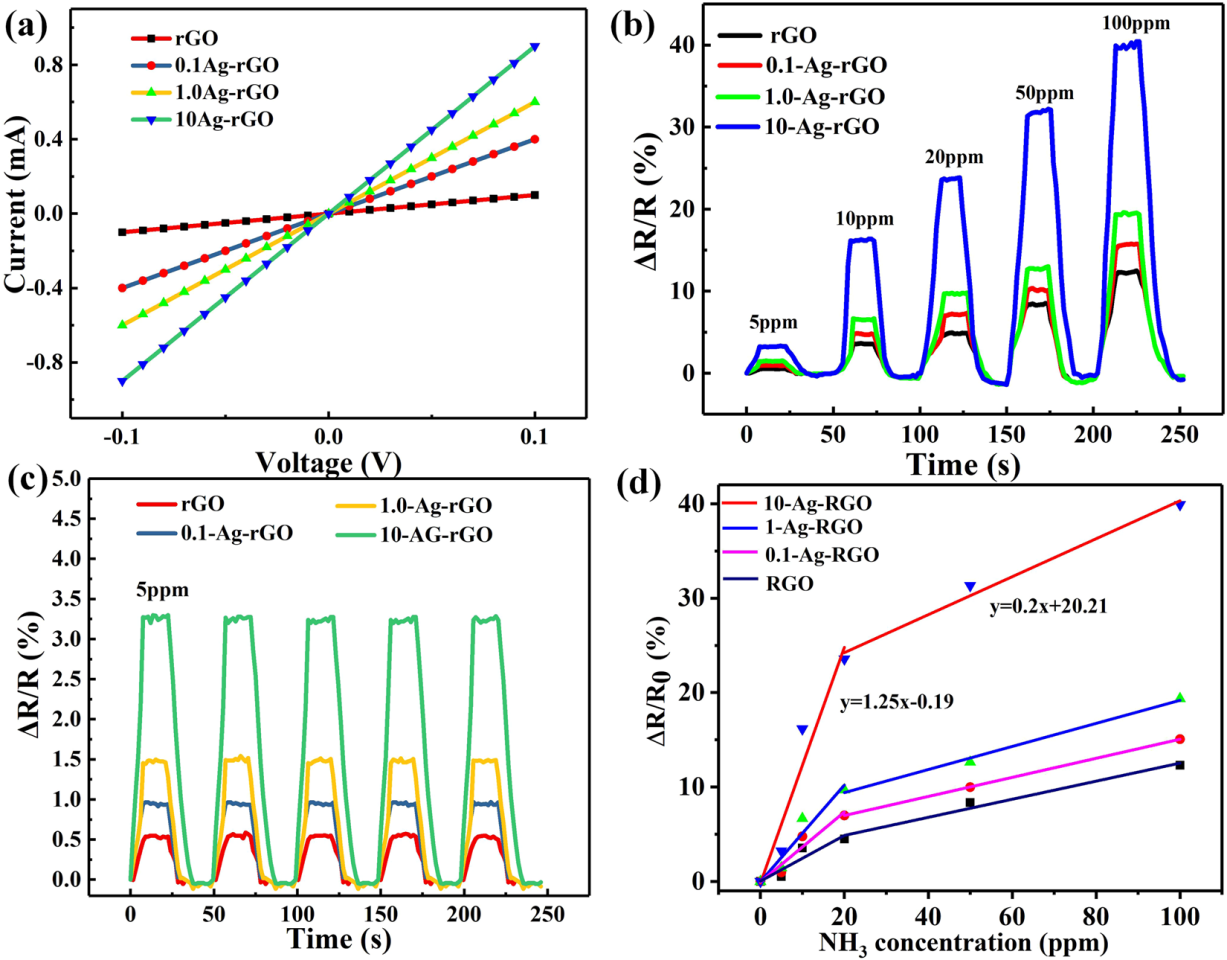
(**a**) I–V characteristic curve for the IDA of different amount of Ag decorated RGO sensing material. (**b**) Normalized resistance changes at different gas concentrations. (**c**) Dynamic response of the sensors exposed to 5 ppm NH_3_. (**d**) The sensitivity of the different sensors.

To evaluate the electrical properties of the Ag-RGO, the Ag-RGO was spray coated on the interdigitated array (IDA) configuration. The IDA electrode was composed of 40-μm-width gold fingers, totalizing 14 pairs. The IDA was formed on PET substrate by magnetron sputtering gold. The distance between IDA fingers is 40 μm. The current-voltage characteristic was measured by HP4145 semiconductor parameters analyzer, which were used to evaluate the electrical conductivity and contact type of the uniformly decorated Ag-RGO on the IDA substrate for various Ag nanoparticles decoration amounts. The current-voltage (I–V) characteristics of them over the voltage range from −0.1 V to 0.1 V, is shown in Fig. 3(a). All devices showed linear I–V characteristics and indicated Ag-RGO is typical ohmic electrical contact with electrodes. It is due to the smooth transfer of charge carriers of the sensing material^31^. With increased of the AgNPs of RGO sheets, the electrical conductivity of the devices enhanced.

Highly pure NH_3_ (99.9%) as a gas source was purchased from Qinlan chemical technology co. LTD. To obtain dry NH_3_, highly pure NH_3_ was passed through a dry tube filled with Ga(OH)_2_ flakes and collected into an aluminum foil air bag. In this work, the gas sensing properties of the Ag-RGO were evaluated by exposition to different concentrations of dry NH_3_. As shown in Fig. 3(b), it is the result of the device sensing material under different concentrations. For the RGO, there is no NH_3_ function sites to attract NH_3_ molecules. In contrast, when the Ag-RGO is exposed to the NH_3_, because of the AgNPs, Ag-RGO shows high sensitivity for NH_3_. To evaluate the dynamic response of the Ag-RGO, IDA were exposed to 5 ppm at room temperature, as shown in Fig. 3(c), it has excellent repeatability and stability. To obtain the sensitivity of the sensor, the response of the sensors at different concentration of NH_3_ were extracted, and then fitted data sectionally. Results of the sensors are shown in Fig. 3(d). When the 10-Ag-RGO sensor exposed to the NH_3_ at 0–20 ppm, the slope is about 1.25. While for the sensor exposed to NH_3_ 20–100 ppm, the slope decreased to 0.2. They are much more sensitive than other sensors at the same concentration of NH_3_. The sensor sensitivity (S) is defined as the following equation:

1 $$S( \% )=\frac{{R}_{{\rm{g}}}-{R}_{0}}{{R}_{0}{\rm{\Delta }}C}\times 100=\frac{{\rm{\Delta }}R}{{R}_{0}{\rm{\Delta }}C}\times 100$$

where R_gas_ (R_g_) and R_0_ are the resistances of the sensor in gas and out of gas, respectively. ΔC is the gas concentration.

### Preparation of sensors

**Figure 4:**
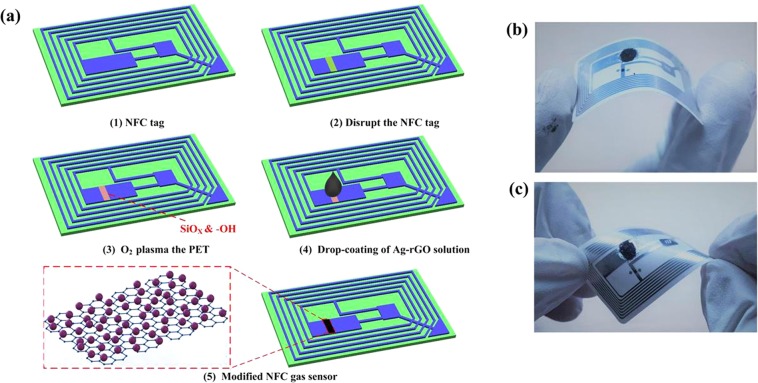
(**a**) Preparation process of NFC tag sensor. (**b**) Drop-coating of Ag-RGO solution onto the NFC tag, which recompleted the LRC circuit. (**c**) NFC sensor tag under twisting.

Figure 4(a) shows a schematic of the preparation process of the near-field communication (NFC) tag sensor. It is a standardized commercial NFC tag (Fudan M1 tag, Chenming Smart Card Technology co. LTD, Guangzhou), with the initial frequency of 13.56 MHz in the first image, and has been widely used in daily life because of its low cost. It is composed simply of an integrated circuit (IC) chip and chip capacitor (C) with an inductance of L on a polyethylene terephthalate (PET) substrate^32^. The NFC tag was disrupted by removing a section of the conductive aluminum. In the second image, and the resonant frequency was changed to 16.78 MHz. To make the surface of PET active, the position that was disrupted was treated by O_2_ plasma as shown in the third image, the plasma power is 120 W and the O_2_ flow rate is 150 sccm. In addition, the processing time is 20 s. This process can generate hydrophilic functional groups on the surface of PET, such as hydroxyl (-OH) or carboxyl (-COOH) groups. Then, the prepared Ag-RGO solutions with different concentrations were drop coated onto the surface of PET by micropipette, the drop volume is 0.5 μL. The LRC loop was reconnected, as shown in the fourth image. The last image shows a schematic of the modified NFC tag sensor. To test the mechanical stability of the sensor, the prepared sensor was bent and twisted, and then kept in an intact state, as shown in Fig. 4(b,c).

## Discussion

**Figure 5:**
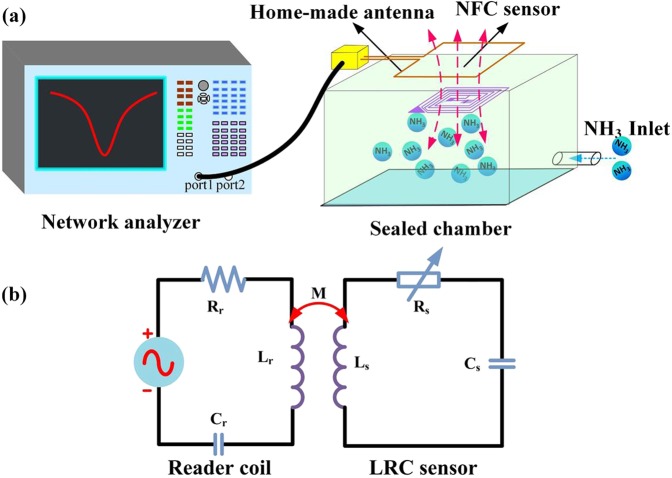
(**a**) Schematic of the test loop. (**b**) Equivalent circuit diagram of the test loop.

A schematic of the NFC tag wireless sensing system for NH_3_ is shown in Fig. 5(a). The sensing system contains a network analyzer (Agilent E5061B), a home-made matched antenna, modified NFC tag, and a sealed chamber. When the network analyzer emitted a swept-frequency signal through the antenna, the sensor tag was activated. Then, the signal was reflected to the antenna, owing to the impedance matching between the antenna and NFC tag, and the antenna resonated with the NFC tag. Meanwhile, the network analyzer received impedance information (phase and amplitude), and changes in the target gas concentration are observed from the impedance information of the antenna. Figure 5(b) shows the equivalent circuit of the gas test device. The antenna terminal contains a signal source, equivalent resistance (R_a_), equivalent inductance (L_a_) and equivalent inductance capacitance (C_a_). The sensor part contains equivalent inductance (L_s_), equivalent capacitance (C_s_), and a variable resistor (R_s_). When the gas sensor was exposed to different concentration of NH_3_, the resistance of the variable resistor of the sensor changed, and the impedance information of the antenna changes accordingly.

According to Kirchhoff’s law, the antenna terminal input impedance (Z_in_) can be expressed as follows^32^:

2 $${Z}_{in}={R}_{a}+j\omega {L}_{a}+\frac{{\omega }^{2}{M}^{2}}{{R}_{s}+j(\omega {L}_{s}-1/\omega {C}_{s})}$$

where ω is the angular frequency and M is the mutual inductance between the antenna and the sensor. The resistance change of the variable resistor in response to NH_3_ causes change in the reflection value (S_11_ parameter) at the resonance frequency, and the simplified formula can be expressed as follows^33^:

3 $${S}_{11}={\frac{{Z}_{in}-{Z}_{0}}{{Z}_{in}+{Z}_{0}}|}_{{Z}_{0}=50{\rm{\Omega }}}$$

When the input impedance Z_in_ in the loop increases as the concentration of NH_3_ increases, the resulting S_11_ value also increases. In the equation (2), the Z_0_ is fixed for all samples.

**Figure 6:**
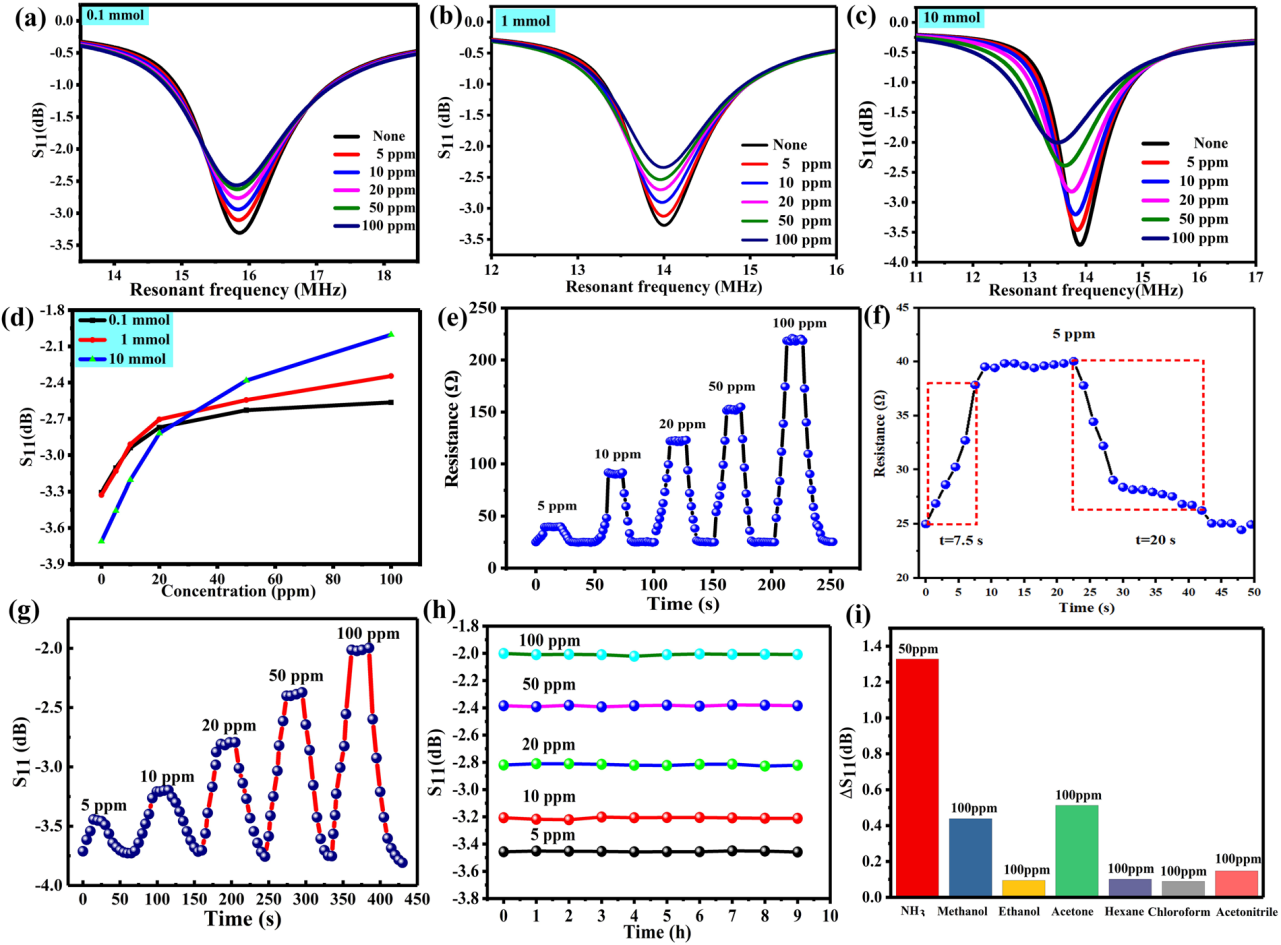
(**a**) Results for the sensor containing 0.1-Ag-RGO. (**b**) Results for the sensor containing 1-Ag-RGO. (**c**) Results for the sensor containing 10-Ag-RGO. (**d**) Reflection characteristic curves for three wireless sensors under different concentrations of ammonia. (**e**) The resistance of 10-Ag-RGO sensor under different concentration of NH_3_. (**f**) The response and recovery time of gas sensor. (**g**) Response of sensors under different concentrations of NH_3_. (**h**) Results of stability tests of sensors under different concentrations of NH_3_. (**i**) The response of prepared sensor to other gases at room temperature.

Figure 6(a–c) show the changes in scattering parameters of different NFC sensors. When there is no NH_3_, the sensor is under the initial state, due to different amount of AgNPs decorated RGO, the initial resistance of the sensor is different and the S_11_ value is also different, the S_11_ absolute value of the 10-Ag-RGO sensor is max. It can be concluded that as the AgNPs increases, the resistance of the sensor decreases. When NFC sensors were exposed to different concentrations of NH_3_ (5–100 ppm), the impedance of the sensor circuit increased gradually. The results in Fig. 6(a,b,c) revealed that the frequencies in Fig. 6(a,b) changed slightly, and only the S_11_ parameter changed. However, in Fig. 6(c), both the S_11_ parameter and the resonant frequency changed, and this is because the change in the impedance of the 10-Ag-RGO sensor circuit was larger than those of the 0.1-Ag-RGO and 1-Ag-RGO circuits. From Fig. 6(d), after extracting the S_11_ parameters at the resonance frequency points of the sensor at different concentrations, it can be seen that the change in the blue line is the most obvious. In addition, the resistance of 10-Ag-RGO sensor was tested under different concentration of NH_3_, as shown in Fig. 6(e). The initial resistance of the sensor is 25 Ω, and the resistance of the sensor increased with the increase of ammonia concentration. The response time of the sensor was defined as the resistance change of the sensor, which was 90% of the maximum value at the ammonia concentration of 5 ppm. Recovery time is defined as the time required for the sensor to recover 90% of the resistance drop after exposure. As shown in Fig. 6(f), the gas sensor possesses fast response time of 7.5 s and recovery time of 20 s, respectively, and the concentration of NH_3_ is 5 ppm. In order to study the wireless response characteristics of NFC gas sensor with different concentration, the NFC sensor was exposed to NH_3_ gas at concentrations of 5, 10, 20, 50, and 100 ppm, respectively. The characteristic curve of the S_11_ parameters with time is shown in Fig. 6(g), it can be seen that sensor has fast response time and recovery time. For practical applications of gas sensor, the fast response, recovery time and good repeatability are essential properties.

The fast response time is attributed to high sensitivity of Ag-RGO nanomaterial. Due to the high specific surface area of RGO, which offers more attachment points and results in more active sites for NH_3_ sensing^34^. NH_3_ is a typical reducing gas, acting as an electrical donor during gas sensing. Due to the catalysis of AgNPs, the sensing ability of RGO increased. In addition, RGO possess carrier mobility (15000 cm^2^ V^−1^S^−1^)^35^. Due to the high carrier mobility of the RGO, it can also act as conducting channel in the gas sensor. Additionally, the S_11_ parameters at the resonant frequency point were extracted. Figure 6(h) shows the repeatability results of NFC sensors at different concentrations of NH_3_ gas. It can be concluded that the sensor has good repeatability. The selectivity of the Ag-RGO based sensor was investigated and the result is presented in Fig. 6(i), the sensor shows high selectivity to NH_3_ compared to other volatile gas, such as ethanol, methanol and acetone. From Fig. 6(i), the response to 50 ppm NH_3_ is much higher than other volatile gases such as methanol, ethanol, acetone, hexane, chloroform and acetonitrile at 100 ppm. These results show that the sensor based on the Ag-RGO possesses an effective selectivity.

Table 1 shows a comparison of the performance of various sensors based on different sensing materials at room temperature. From the table, there are a few sensors’ MDL superior to the sensor in this work. However, most sensors’ response time and recovery time are too long for the poisonous gas sensors. Compared to other gas sensors, our gas sensor shows better performance in MDL, response time and recovery time. In the Table 1, the MDL is minimum detectable level, T_res_ is response time; T_rec_ is recovery time.

**Table 1:** Comparison of sensor perforamance for the Ag-RGO with other sensors.

Sensing material	T (°C)	MDL (ppm)	Sensitivity (%)	Tres (s)	Trec (s)	connection	Ref.
ZnO	RT	400	—	120–180	600–1200	Wire	^41^
RGO	RT	0.4	1.5	600	600	Wire	^42^
Au-CNT	RT	50	0.03	84	216	Wire	^43^
ZnO-RGO	RT	0.05	0.384	50	250	Wire	^44^
Pd/SnO2/RGO	RT	5	0.124	420	3000	Wire	^45^
Carbon black	RT	151	—	150	120	RFID	^46^
Ag-RGO	RT	5	1.25	7.5	20	RFID	This work

**Figure 7:**
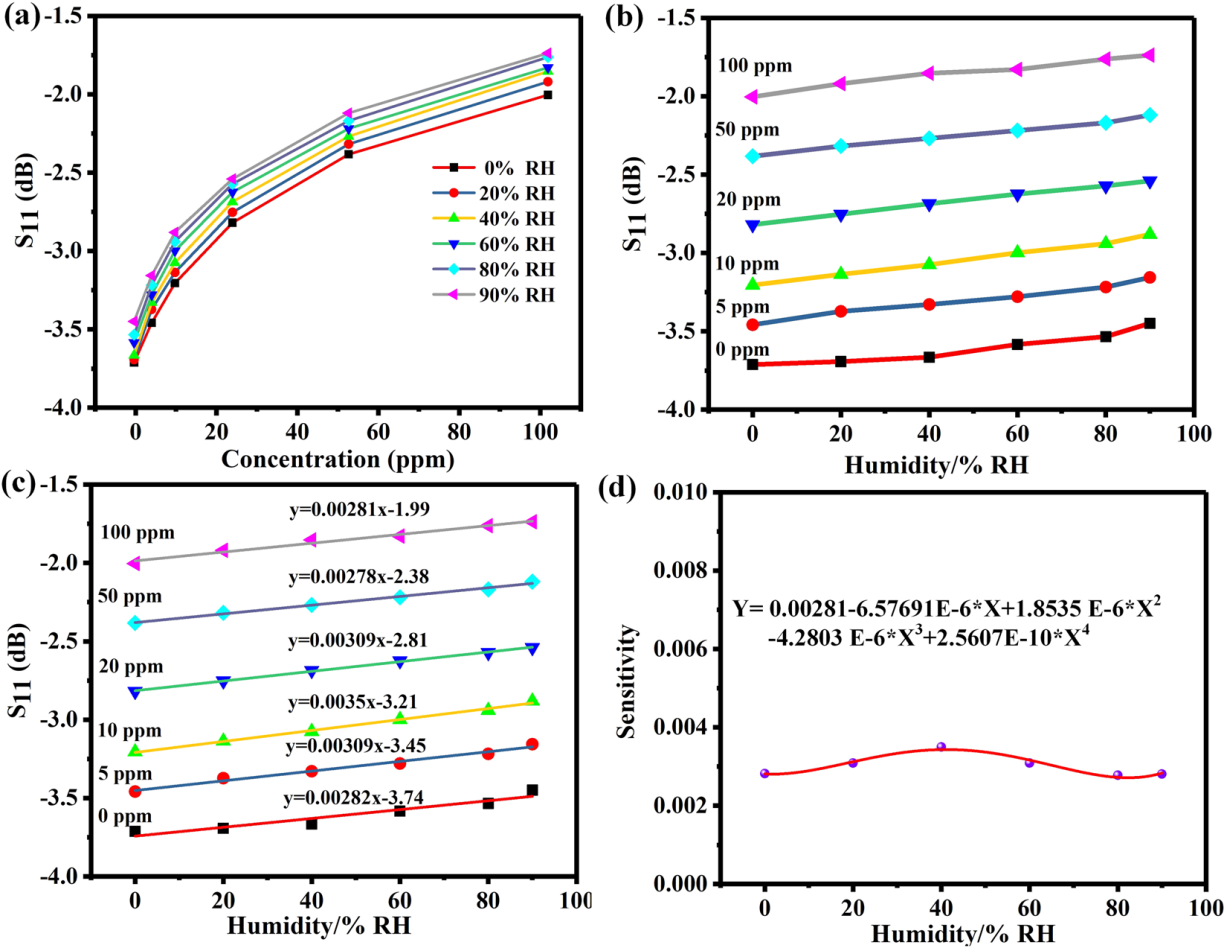
(**a**) S_11_ parameters extracted from different concentration of NH_3_ at relative humidity 0–90% RH. (**b**) S_11_ parameters extracted under humidity 0–90% RH at concentration of NH_3_ 0–100 ppm. (**c**) Linear fitting of the values of S_11_. (**d**) The quartic polynomial fitting curve of the average humidity sensitive.

To study the effect of humidity, the sensor was exposed to different concentration of NH_3_ (5 ppm, 10 ppm, 20 ppm, 50 ppm, 100 ppm). When the relative humidity of the environment increased, the S_11_ of the sensor decreased, as shown in Fig. 7(a). By extracting the S_11_ of the sensor at different humidity, results of the sensor are shown in Fig. 7(b). It can be found that the response of the sensor increased as the increased of relative humidity. In addition, we performed linear fitting of extracted data under different humidity, and obtained influence coefficient of humidity on sensor response at different concentration of NH_3_ which is the slope of fitted line, as shown in Fig. 7(c). Finally, the influence coefficient curves of different humidity on the sensor are obtained, as shown in Fig. 7(d). Hence, the sensitivity coefficient can be calculated when the humidity value was substituted into the quartic polynomial fitting curve.

As is well known, noble metals are introduced as catalyst usually^17^, metal decorated on the surface of a sensing film can improve the sensing ability for the objective gas molecule dramatically. However, it is not reasonable to attribute the gas-sensing ability simply to increases in the specific surface area. When the Ag-RGO was exposed to the air, oxygen molecules adsorbed on their surface, due to the catalytic action of AgNPs, O_2_ molecules trapped electrons and they transform into O_2_^−^ as shown in eq. (3)^36^. NH_3_ as a typical reducing gas, is always an electron donor. When NH_3_ molecules were absorbed on the surface of AgNPs, the sensing mechanism can be described as equations (4–8)^37^.

4 $${{\rm{O}}}_{2}({\rm{g}})+{{\rm{e}}}^{-}\to {{\rm{O}}}_{2}{({\rm{ads}})}^{-}$$

5 $$4{{\rm{NH}}}_{3}({\rm{g}})+3{{\rm{O}}}_{2}{({\rm{ads}})}^{-}\to 2{{\rm{N}}}_{2}+6{{\rm{H}}}_{2}{\rm{O}}+3{{\rm{e}}}^{-}$$

6 $$4{{\rm{NH}}}_{3}({\rm{g}})+5{{\rm{O}}}_{2}{({\rm{ads}})}^{-}\to 4{\rm{NO}}+6{{\rm{H}}}_{2}{\rm{O}}+5{{\rm{e}}}^{-}$$

7 $$2{\rm{NO}}+2{{\rm{O}}}_{2}({\rm{g}})\to 2{{\rm{NO}}}_{2}$$

8 $${{\rm{NO}}}_{2}+{{\rm{e}}}^{-}\to {{\rm{NO}}}_{2}{({\rm{ads}})}^{-}$$

9 $${{\rm{NO}}}_{2}+{{\rm{O}}}_{2}({\rm{g}})+2{{\rm{e}}}^{-}\to 2{{\rm{NO}}}_{3}{({\rm{ads}})}^{-}$$

**Figure 8:**
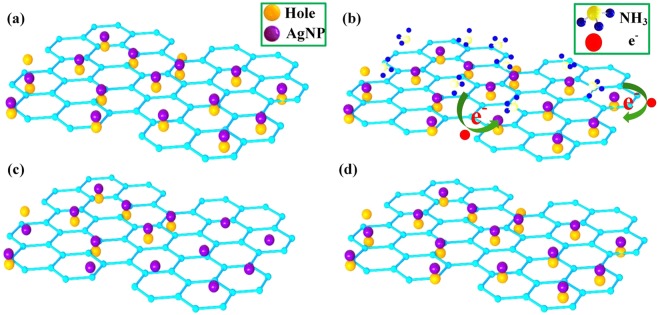
Schematic diagram of the principle of NH_3_ gas sensing by reduced graphene oxide: (**a**) Ag-RGO in air. (**b**) Ag-RGO accepts electrons in an NH_3_ atmosphere. (**c**) The number of holes of graphene decreases. (**d**) Ag-RGO returns to the original state.

For gas sensing, electronic sensitization is a widely accepted theory^38^. The Fermi energy level of eigenstate graphene is near the Dirac point^39^, when graphene adsorbs water and oxygen molecules under ambient conditions, it shows the characteristics of a typical p-type semiconductor, and holes act as the major carriers^40^, as shown in Fig. 8(a). As a typical reducing gas, when NH_3_ was adsorbed to AgNPs decorated RGO, electrons were transferred from NH_3_ to the graphene and consumed the holes of graphene, as shown in Fig. 8(b), resulting in the resistance increases shown in Fig. 8(c). When the sensor was re-exposed to air, the number of holes of RGO increased quickly, and the electrical conductivity of Ag-RGO returned to the original value, as shown in Fig. 8(d). It is reasonable to conclude that AgNPs enhanced the capability of graphene to gain electrons. In this system, the AgNPs greatly enhanced the specific surface area of RGO, while the sensitivity of graphene to NH_3_ was improved.

## Conclusion

In summary, we have prepared a NFC-based wireless gas sensor, based on a modified commercial NFC tag and the Ag-RGO as a sensitive material. It possesses a fast response time (7.5 s) and recovery time (20 s). To test the properties of the sensor, a platform for NH_3_ testing was set up. The results indicated that the prepared wireless sensor showed good performance in the detection of NH_3_ when the concentrations ranged from 5 ppm to 100 ppm at room temperature. Then it also possesses good recoverability and stability at different concentration. Therefore, the proposed wireless passive sensor has the potential for application in future wearable gas sensors.

## Methods

GO sheets were obtained from natural graphite flakes (99%, Aldrich) using the modified Hummers and Offman method. 50 mg of GO was dispersed in 50 mL of deionized water via sonication treatment for 60 min at room temperature, and resulting GO sheets were stripped to be single sheet structure, affording a brown liquid, which contributed to AgNPs adsorption on the surface of GO sheet. Then, 0.01, 0.1, or 1 mmol of a AgNO_3_ solution was added to the graphene solution, followed by NaBH_4_ as the reducing agent, and the resulting mixture was stirred at a speed of 1500 rpm at 25 °C for 1 h, producing Ag-GO nanoparticles. This procedure was performed in a photo area, which can prevent the decomposition of AgNO_3_. Then, 10 mL of a solution of ascorbic acid, which is an innocuous green reducing agent, was introduced to the solution of silver-modified GO. In order to enhance the reduction effect, the reaction was stirred at a speed of 1500 rpm at 95 °C for 1 h. In order to remove impurities from the solution, it was washed repeatedly using deionized water, and then filtered. Ag-rGO powders were obtained after drying in a vacuum oven at 25 °C for 24 h.
